# Clinical progress of anti-angiogenic targeted therapy and combination therapy for gastric cancer

**DOI:** 10.3389/fonc.2023.1148131

**Published:** 2023-06-12

**Authors:** Donghan Xu, Yehao Luo, Peng Wang, Jiaxin Li, Linrui Ma, Jie Huang, Hao Zhang, Xiaoman Yang, Liqi Li, Yuhong Zheng, Gang Fang, Peiyu Yan

**Affiliations:** ^1^ Faculty of Chinese Medicine, Macau University of Science and Technology, Macao, Macao SAR, China; ^2^ School of Second Clinical Medicine, Guangzhou University of Chinese Medicine, Guangzhou, China; ^3^ Guangxi Key Laboratory of Applied Fundamental Research of Zhuang Medicine, Guangxi University of Chinese Medicine, Nanning, China; ^4^ State Key Laboratory of Quality Research in Chinese Medicines, Macau University of Science and Technology Zhuhai MUST Science and Technology Research Institute, Macau University of Science and Technology, Macao, Macao SAR, China

**Keywords:** gastric cancer, anti-angiogenic, tyrosine kinase inhibitor, response biomarker, clinical progress

## Abstract

The incidence of gastric cancer is increasing year by year. Most gastric cancers are already in the advanced stage with poor prognosis when diagnosed, which means the current treatment is not satisfactory. Angiogenesis is an important link in the occurrence and development of tumors, and there are multiple anti-angiogenesis targeted therapies. To comprehensively evaluate the efficacy and safety of anti-angiogenic targeted drugs alone and in combination against gastric cancer, we systematically searched and sorted out relevant literature. In this review, we summarized the efficacy and safety of Ramucirumab, Bevacizumab, Apatinib, Fruquintinib, Sorafenib, Sunitinib, Pazopanib on gastric cancer when used alone or in combination based on prospective clinical trials reported in the literature, and sorted response biomarkers. We also summarized the challenges faced by anti-angiogenesis therapy for gastric cancer and available solutions. Finally, the characteristics of the current clinical research are summarized and suggestions and prospects are raised. This review will serve as a good reference for the clinical research of anti-angiogenic targeted drugs in the treatment of gastric cancer.

## 1 Introduction

Gastric cancer (GC) is one of the most common causes of cancer death and ranks third in cancer-related death worldwide (1, 2). The survival rate of GC has improved in recent years, but its mortality rate still accounts for 23.4% of malignant tumors (3), and the 5-year survival rate is less than 10% (4). For the lack of effective screening methods, most GC patients are diagnosed at advanced stage, resulting in poor prognosis with mPFS less than 12 months (5).

The treatment of GC mainly includes surgery, radiotherapy, chemotherapy and biological targeted therapy. Endoscopic mucosal dissection is the main treatment for early GC (6), while surgical treatment (e.g. total gastrectomy, distal gastrectomy, proximal gastrectomy) is the first choice for GC (7) and complete resection is the only curative treatment that may cure GC (8). However, despite aggressive surgical intervention, more than 50% of patients with curative resection experienced disease recurrence in the form of metastatic disease (9). The development of metastatic disease is almost fatal. Despite the advances in chemotherapy regimens for GC, the efficacy is still unsatisfactory and the drug resistance of patients is extremely high (10). Therefore, the research focus is to develop more effective and personalized treatment regimens to prolong survival time and improve quality of life of advanced GC patients.

Angiogenesis is the formation of blood vessels form existing ones and it is also the basis of tumor proliferation, invasion, and metastasis in advanced GC (11). Previous studies have shown that serum VEGF levels of advanced GC patients are higher than those of healthy individuals (12). In GC, tumor cells and stromal cells produce various angiogenic factors, such as vascular endothelial growth factor (VEGF), interleukin-8 (IL-8), and platelet-derived endothelial cell growth factor (PD-ECGF) (13). These factors stimulate the proliferation and migration of endothelial cells, which induces the formation of new capillaries in the tumor microenvironment (14). Angiogenesis is co-regulated by pro-angiogenic and anti-angiogenic factors (15). MicroRNAs (miRNAs) are small non-coding RNAs and they bind to mRNAs to regulate the expression of genes involved in angiogenesis (16). It has been verified that some miRNAs play a role in GC angiogenesis by targeting different angiogenic factors or pathways (17). For example, miR-126 can inhibit GC angiogenesis by suppressing VEGF and its receptor VEGFR2 (18). MiR-34a can inhibit GC angiogenesis by targeting PD-ECGF and ANG2 (19). MiR-221 can promote GC angiogenesis by inhibiting the anti-angiogenic factor thrombospondin-1 (TSP-1) (20). The microenvironment in the gastric mucosa may also affect the angiogenic phenotype of GC as chronic inflammation, hypoxia, and acidosis can upregulate the expression of angiogenic factors and receptors (21). Angiogenesis is an important hallmark of malignancy, thus inhibition of this process has become a hallmark of biological anticancer therapies for solid tumors in the contemporary world (22). Angiogenesis inhibitors have entered various stages of clinical trials and are widely used in the clinic, but a summary of the successes and problems encountered in current clinical studies is lacking.

In this review, in order to provide more personalized treatment plans for GC patients and provide a reliable theoretical basis for the treatment of GC with anti-angiogenic drugs, we systematically expounded the mechanism of angiogenesis on GC and summarized the results of registered clinical trials of anti-angiogenic targeted drugs for GC, as well as the response biomarkers of these drugs. Finally, based on the review, we also discussed the problems in the current research and the direction of future clinical research.

## 2 Angiogenesis mechanism of gastric cancer

The occurrence and development of tumors depend on angiogenesis, and new blood vessels promote tumor invasion and metastasis (23). In the process of angiogenesis, a variety of factors are involved in the regulation. Vascular endothelial growth factor (VEGF) is considered to be the strongest pro-angiogenic growth factor (24), and its receptor (vascular endothelial growth factor receptor, VEGFR) has become a hotspot for research in recent years and great progress has been made (25).

The VEGF family includes vascular endothelial growth factor A (VEGF-A), vascular endothelial growth factor B (VEGF-B), vascular endothelial growth factor C (VEGF-C), vascular endothelial growth factor D (VEGF-D), vascular endothelial growth factor E (VEGF-E) and placental growth factor (PIGF), whose receptors include 3 tyrosine kinase receptors vascular endothelial growth factor receptor 1 (VEGFR-1, also known as Flt-1), vascular endothelial growth factor receptor 2 (VEGFR-2, also known as KDR/Flk-1), vascular endothelial growth factor receptor 3 (VEGFR-3, also known as Flt-4), whose receptors bind to VEGF with high affinity (26).

VEGF receptors are transmembrane tyrosine kinases that activate various angiogenic pathways upon ligand binding and receptor dimerization (27). VEGF-A is the member of the VEGF family that is most closely related to angiogenesis (28). Different isoforms of VEGF-A have different functions depending on their binding affinity and specificity for the receptors (29). For example, both VEGF-A121 and VEGF-A165 bind to VEGFR-1 and VEGFR-2, but interestingly, VEGF-A121 has a higher affinity for VEGFR-2 and is more potent in inducing endothelial cell proliferation and migration than VEGF-A165 (30). On the other hand, VEGF-A189 and VEGF-A206 mainly bind to VEGFR-1 with a lower angiogenic activity than VEGF-A121 and VEGF-A165 (31). Ligand-receptor binding is the basic step for receptor activation and subsequent signal transduction (32). VEGF-A binds to both VEGFR-1 and VEGFR-2, but mainly signals through VEGFR-2 (33). The receptor most closely related to tumor angiogenesis is VEGFR-2 (13). VEGF-B and PlGF only bind to VEGFR-1 and regulate its activity (34). VEGF-C and VEGF-D bind to both VEGFR-2 and VEGFR-3, but mainly signal through VEGFR-3 (35). VEGFR-3 is the specific receptor for lymphatic growth factors VEGF-C and VEGF-D, which regulates the function of both vascular and lymphatic endothelial cells during embryonic development (36).

The function of VEGF in tumor angiogenesis mainly lies in three aspects. First, VEGF is a homodimeric glycoprotein encoded by a single gene, which can directly stimulate the movement, proliferation and division of vascular endothelial cells, increase the permeability of micro-vessels to promote the assimilation of cadherin and reduce the intercellular adhesion (37). VEGF is closely related to nitric oxide (NO) and can reduce endothelial nitric oxide synthetase (NOS) activity (38), reduce vascular tension to increase microvascular permeability, which is conducive to the extravasation of fibrinogen and other plasma proteins, and become the basis of tumor neovascularization network formation and accelerate tumor hematogenous metastasis (39). Second, VEGF changes the activation of endothelial cells, and induces the expression of a series of endothelial cell genes from different sources under hypoxic conditions (40), including the expression of procoagulant factor, plasminogen activator inhibitor-1 (PAI-1), matrix metalloproteinase (MMP), interstitial collagenase and tissue factor, to degrade the extracellular matrix around blood vessels (41) to promote the release of pro-angiogenic factors stored in the extracellular matrix (42) thus inducing vascularization. Third, VEGF is a mitogen of endothelial cells, which activates the MAPK signaling pathway to stimulate the mitosis of endothelial cells and promote the proliferation and deformation (43); VEGF binds to VEGFR-1 (also known as Flt1) and phosphorylates Flt1 (33). Phosphorylated Flt1 cannot significantly promote the proliferation of endothelial cells, but it can activate the actin reorganization induced by P38-MAPK of the mitogen-activated protein kinase (MAPK) family and promote the migration of endothelial cells (44), while PIGF binds to Flt-1 to increase endothelial cell proliferation by activating p38 MAPK (45); VEGF is rapidly phosphorylated upon binding to VEGFR-2, and the phosphorylation activates multiple signal transduction molecules (46), including P38 - MAPK, PI3K, Akt/PKB, PKC, Ras GAP, Raf -1, MEK, ERK. Phosphorylated VEGFR-2 promotes mitosis and proliferation of endothelial cells by activating the MAPK pathway and PKC-MAPK bypass (47). VEGF is phosphorylated after binding to VEGFR-3 and this activates p42/p44 MAPK transduction through the Ras-independent pathway and promotes the proliferation of lymphatic endothelial cells (48). Phosphorylation of VEGFR-3 can also activate PI3K/Akt, thereby transducing survival signals in lymphatic endothelial cells and vascular endothelial cells to prevent cell apoptosis (49).

**Figure 1:**
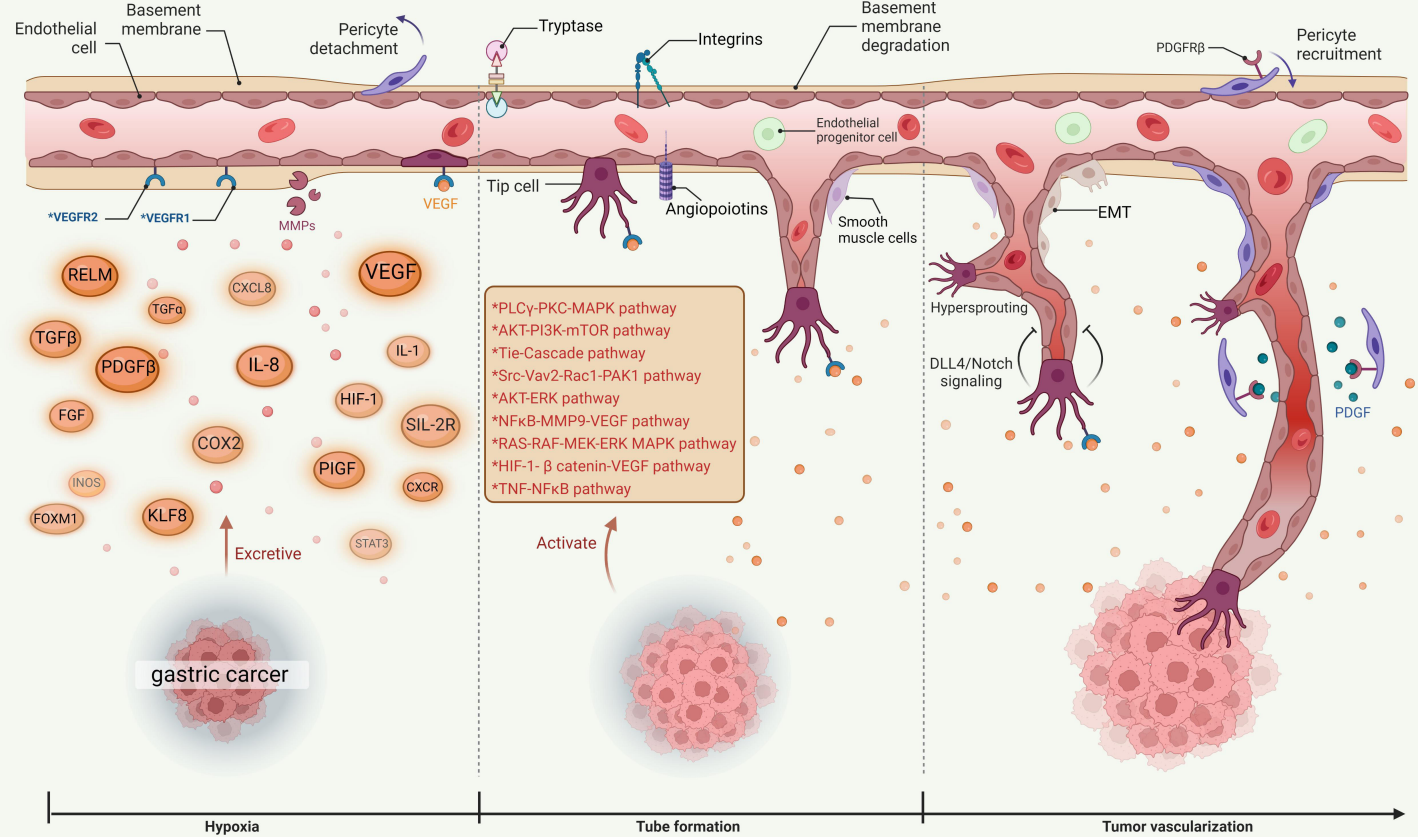
Diagram of angiogenesis in GC.

VEGF is closely related to GC. Studies have confirmed that the expression level of VEGF in cancer tissue and serum of patients with GC can be a reliable indicator of GC occurrence, development, metastasis and prognosis. Some studies (50–52) compared the expression level of VEGF in GC tissue and normal gastric mucosal tissue, and the results indicated that the expression level of VEGF in GC tissue was higher than that in normal gastric mucosal tissue and was related to pathological type, TNM system, and lymph node metastases (53), which suggests that VEGF may be a potential diagnosis and treatment indicator for the occurrence and development of GC. The VEGF in the serum of patients with GC mainly comes from the primary lesion, while VEGF also recruits effector cells such as endothelial cells, hematopoietic stem cells, osteoblasts and osteoclasts in the bone marrow to the site of neovascularization, thereby forming the embryonic form of new blood vessels, increasing VEGF expression in peripheral blood (54). The high expression of VEGF is an important turning point of angiogenesis in GC. The level of serum VEGF correlates with tumor type and infiltration depth (55), and also lymph node metastasis (56), hematogenous metastasis (57, 58) and early recurrence (59). VEGF promotes the maturation and stability of the neovascular bed, and it not only provides key nutrients for tumor growth, maintains and promotes tumor growth, but also facilitates tumor metastasis as a tubular channel (60) (See **Figure 1** for more details).

## 3 Current monoclonal antibodies for angiogenesis treatment in gastric cancer

### 3.1 Ramucirumab

Ramucirumab is an intravenously administered fully human IgG1 monoclonal antibody derived from phage display technology (61). Ramucirumab inhibits angiogenesis, and it binds to the end of the extracellular domain with high affinity, which induces spatial overlap and conformational changes in the receptor that ultimately prevents ligand binding to VEGFR-2, thereby inhibiting downstream signaling (62). VEGFR-2 is the primary receptor responsible for the spectrum of VEGF -induced biological changes that drive many cancers, including changes in vascular structure and function, proliferation and migration (63). Unlike clinically approved angiogenesis inhibitors, Ramucirumab has specificity and potently inhibits VEGFR-2 (64).

Ramucirumab is the first molecular-targeted drug (65) for clinical single intervention. Its clinical application for GC treatment is shown in **Table 1**. The earliest study was a phase 3 REGARD trial (73) conducted in 2009, which involved 355 patients with GC or gastroesophageal junction adenocarcinoma who underwent first-line platinum- or fluoropyrimidine-containing chemotherapy. The patients were divided into 2 groups and given ramucirumab and placebo interventions and the results showed that the ramucirumab group had a longer survival period. It is worth noting that the incidence of hypertension in the ramucirumab group was higher than that of the placebo group. An open-label, non-random phase 2 clinical trial in Japan showed (74) that the median overall survival (mOS) of the ramucirumab group was 8.6 months; median progression-free survival (mPFS) was 6.6 months; 12-week progression-free survival rate (12-week PFS rate) was 23.8%, and diarrhea, loss of appetite, high blood pressure, gastric bleeding and protein urine and other adverse events were reported. Taking the REGARD trial as a reference, ramucirumab showed clinical activity and controllable safety in this study.

**Table 1:** Clinical trials of anti-angiogenic targeting monoclonal antibodies in the treatment of GC.

Monoclonal antibodies	Country/Number of Enrollments	NCT number/Phase/Status	Patient population	Treatment arm	Primary Efficacy outcome	Treatment-related adverse events	References
Ramucirumab	United States/355	NCT00917384/3/Completed	Patients with GC with disease progression after first-line platinum- or fluoropyrimidine-combined chemotherapy	Ramuciridine vs placebo	mOS 5.2months vs 3.8months, HR = 0.776, 95%CI0.603-0.998, P=0.0473. mPFS 2.1 months vs 1.3 months, HR = 0.483, P<0.0001. 12-week PFS rate 40.1% versus 15.8%, HR=24.2, p<0.0001	Hypertension	(35)
Ramucirumab	Japan/36	NCT01983878/2/Completed	Patients with GC with disease progression after first-line chemotherapy	Ramucirumab	mOS 8.6 months, mPFS 6.6 months, 12-week PFS rate 23.8%	Hypertension, bleeding, proteinuria, diarrhea, decreased appetite, intestinal obstruction	(36)
Ramucirumab, paclitaxel, placebo	China/440	NCT02898077/3/Completed	Adult patients with GC for whom prior fluoropyrimidine/platinum chemotherapy failed	Ramucirumab + paclitaxel vs placebo + paclitaxel	mPFS 4.14 months vs 3.15 months, HR = 0.765, P =0.0 184. mOS 8.71 months vs 7.92 months, HR = 0.963, p=0.74 26.	Decreased neutrophil count	(66)
Ramucirumab, Capecitabine, cisplatin, placebo, 5- fluorouracil	United States/645	NCT02314117/3/Completed	GC without first-line chemotherapy	Ramucirumab + cisplatin + 5- fluorouracil vs placebo + cisplatin + 5- fluorouracil	mPFS 5.72 months vs 5.39 months, HR = 0.753, P = 0.0106.	Decreased neutrophil count, anemia, hypertension, vomiting, diarrhea	(67)
Ramucirumab, paclitaxel, placebo	United States/665	NCT01170663/3/Completed	Patients with GC who have previously received first-line therapy	Ramucirumab +paclitaxel vs placebo + paclitaxel	mOS 9.6 months vs 7.4 months, HR = 0.807, P=0. 0169. mPFS 4.4 months vs 2.9 months, HR = 0. 635, P<0.0001. mTTP 5.52 months vs 3.02 months, HR = 0. 596; P <0.0001. ORR 27.9% vs 16.1%	Neutropenia, leukopenia, hypertension, fatigue, anemia, abdominal pain	(68)
Ramucirumab, irinotecan, leucovorin, 5- fluorouracil, paclitaxel	USA/29	NCT03081143/2, 3/Completed	Patients with GC who have previously received first-line therapy	FOLFIRI plus ramucirumab	ORR 23%, mOS 13.4 months, mPFS 6 months, 6-month OS rate 90%, 12-month OS rate 41%	Fatigue, diarrhea, anemia, neutropenia	(69)
Ramucirumab, s-1, oxaliplatin, paclitaxel, placebo	United States/189	NCT02539225/2/Completed	Patients with GC who have previously received first-line therapy	s-1/oxaliplatin + ramucirumab vs S-1/oxaliplatin + placebo	mPFS 6.34 months vs 6.74 months, ORR 58.2% vs 50%	Neutropenia, vomiting, anemia, decreased appetite	(70)
Bevacizumab, capecitabine, cisplatin, placebo, 5- fluorouracil	United States/774	NCT00548548/3/Completed	Histologically confirmed GC with inoperable, locally advanced, or metastatic disease.	Bevacizumab + chemotherapy vs placebo + chemotherapy	mOS 12.1 months vs 10.1 months, HR = 0.87, P= 0.10 02. mPFS 6.7 months vs 5.3 months, HR = 0.80, P=0.00 37. ORR 46.0% vs 37.4%, P=0.0 315	neutropenia, anemia, decreased appetite	(71)
Cisplatin, irinotecan, bevacizumab	USA/47	-/2/Completed	Pathologically confirmed GC patients were required to have prior untreated metastatic or unresectable disease.	Bevacizumab + cisplatin + irinotecan	ORR 65%, mTTP 8.3 months, mOS 12.3 months	Hypertension, GI perforation, GI bleeding, thromboembolism	(72)

FOLFIRI, irinotecan + 5- fluorouracil + calcium folinate; s-1, Tegafur Gimeracil and Oteracil.

As Ramucirumab alone showed great efficacy in the second-line treatment of GC or gastroesophageal junction adenocarcinoma, the US Food and Drug Administration (FDA) approved ramucirumab for the treatment of GC or gastroesophageal junction adenocarcinoma in 2014 (61). A US multicenter, double-blind, randomized phase 3 RAINBOW trial was conducted in 170 centers in 27 countries in North America, South America, Asia and Australia (66), and it also recruited patients with GC or gastroesophageal junction adenocarcinoma. Patients in the test group (330/665) received ramucirumab plus paclitaxel, and patients in the control group (335/665) received placebo plus paclitaxel. The results showed that the median overall survival and median progression-free survival of the ramucirumab paclitaxel group were better than those of the placebo paclitaxel group (mOS 9.6 months vs 7.4 months; mPFS 4.4 months vs 2.9 months) (68). Another RAMIRIS trial compared the safety and efficacy of FOLFIRI plus ramucirumab with paclitaxel plus ramucirumab for the same patients, and the results showed that FOLFIRI plus ramucirumab has a relative advantage in terms of the objective response rate (ORR), mPFS, and mOS (69). Ramucirumab has become the world’s first molecularly targeted drug proven to be effective in the second-line treatment of GC or gastroesophageal junction adenocarcinoma combined with chemotherapy, providing a new option for such patients (75).

By comparison, in the RAINBOW trial, both Japanese and Western patients had improved mPFS and ORR after ramucirumab combined with paclitaxel and had similar safety profiles. However, in the evaluation of adverse reactions, the incidence of neutropenia in Japanese patients was higher than that in Western patients (76, 77) Thus, a randomized, multicentre, double-blind phase 3 RAINBOW-Asia trial (78, 79) (a bridging study similar to RAINBOW) was initiated, in which patients were randomized to receive ramucirumab plus paclitaxel (n=294) or placebo plus paclitaxel (n=146), the primary outcome was (mPFS 4.14 months vs 3.15 months, mOS 8.71 months vs 7.92 months), and the most common adverse reaction was also decreased neutrophil count (54% vs 39%). In 2021, Eli Lilly and Company announced that the RAINBOW-Asia trial had reached its pre-specified research priority, and the results, together with the RAINBOW results, supported ramucirumab in combination with paclitaxel as a second-line therapy for GC or gastroesophageal junction adenocarcinoma (80).

Based on the efficacy and safety of ramucirumab, researchers began to explore the efficacy and safety of ramucirumab combined with different chemotherapy drugs for the patients. A phase 3 RAINFALL study of ramucirumab plus a fluoropyrimidine and cisplatin or placebo plus a fluoropyrimidine and cisplatin (67) showed statistical significance in the primary analysis of mPFS, but the results were not confirmed in a sensitivity analysis of progression-free survival by central independent review because the results of mOS were not statistically significant. Therefore, it is not recommended to add ramucirumab to cisplatin plus fluoropyrimidine chemotherapy as first-line therapy. An East Asian multicenter, double-blind, randomized, phase 2 RAINSTORM trial of mPFS (70) suggested that the addition of ramucirumab into fluoropyrimidine and cisplatin did not prolong mPFS or mOS, which means not all chemotherapy regimens combined with ramucirumab can increase the efficacy.

### 3.2 Bevacizumab

Bevacizumab is the first VEGF monoclonal antibody (81). Bevacizumab is a recombinant humanized monoclonal IgG1 antibody that can bind to VEGF. It can bind to VEGF-A to inhibit the activity of VEGF and block its interaction with VEGFR-1 and VEGFR-2 (82), including endothelial cell enhanced vascular permeability activity, mitogenic activity and other pro-angiogenic activities, to inhibit tumor angiogenesis (81). Bevacizumab regresses tumor vessels and normalizes remaining tumor vessels while inhibiting neovascularization or recurrent angiogenesis (83). As one of the earliest therapies targeting the tumor microenvironment (84), bevacizumab has been used as a targeted therapy drug for various cancers (85). Several studies have demonstrated that bevacizumab exhibits modest antitumor activity in a wide range of malignancies when used in combination with chemotherapy (86).

In recent years, progress has been made in the treatment of metastatic colorectal cancer, NSCL, metastatic breast cancer, ovarian cancer, cervical cancer and other cancers with bevacizumab (87). Bevacizumab was originally approved for the treatment of metastatic colorectal cancer in the United States (US) and the European Union (EU) in 2004 and 2005, respectively (88). Many clinical studies proved the efficacy of bevacizumab (See **Table 1** for more details).

GC treatment with bevacizumab has been researched recently (89). Some studies suggested that bevacizumab was not recommended as a perioperative treatment for patients with resectable GC (90). However, there is no unified conclusion on the predictive indicators of the efficacy of bevacizumab at present, and it is still impossible to confirm which group of people is suitable for bevacizumab treatment. A multicenter trial with small samples evaluating bevacizumab combined with chemotherapy in the treatment of GC and gastroesophageal junction adenocarcinoma was conducted in the United States. The combined regimen (72) had an ORR of 65%, a median time to disease progression (mTTP) of 8.3 months, and an mOS of 12.3 months. Compared with historical controls, mTTP improved by 75%. The toxicity profile included hypertension, gastrointestinal perforation, gastrointestinal bleeding, and thromboembolic events, and was not different from other bevacizumab-containing regimens. It is suggested that we optimize the use of bevacizumab in the treatment of GC (91).

The following AVAGAST was an international clinical study involving patients from Europe, the United States, Korea, and Japan. Chemotherapy in combination with or without bevacizumab was taken as first-line treatment for patients with GC (71). The included patients received subsequent chemotherapy after disease progression, and the results showed that the ORR, mOS and mPFS of the combination group were longer than those of the chemotherapy group. In baseline comparison, poor performance status, liver metastases, and larger tumors were most common in Eastern Europe/South America and the least common in Japan. Although the study did not meet the expected goals, the addition of bevacizumab to chemotherapy increased the mPFS and ORR of the first-line treatment of GC, and also showed geographical differences, with the greatest survival benefit in American patients and almost no survival benefit in Asian patients.

Considering the influence of geographical factors in AVAGAST, Chinese researchers designed an AVATAR (92)trial (randomized, double-blind, phase III) similar to AVAGAST for Chinese patients with GC. The patients in the experimental group received bevacizumab combined with capecitabine-cisplatin. There was no difference in mOS between the experimental and placebo groups, and mPFS was also similar. Safety findings were identical to those of a previous US trial of bevacizumab (AVAGAST); bevacizumab plus capecitabine-cisplatin was well tolerated, with 60% of bevacizumab-treated patients and68% of placebo-treated patients reported grade 3-5 adverse events (AEs). It can be seen that the AVATAR trial did not bring survival benefits to Chinese patients with GC.

## 4 Tyrosine kinase inhibitors currently used to treat gastric cancer angiogenesis

### 4.1 Apatinib

Apatinib is a small-molecule VEGFR-2 tyrosine kinase inhibitor independently developed in China. It is the first small-molecule targeted drug proven to improve the survival of GC (93)(See **Table 2** for more details). Apatinib was approved by the China Food and Drug Administration (CF-DA) in 2014 for the treatment of third-line and above-advanced GC (124), making it a new option for patients with GC who failed in second-line treatment (125). Meanwhile, studies have shown that the overall response rate of apatinib for advanced GC is 42% (126).

**Table 2:** Clinical trials of anti-angiogenic targeted tyrosine kinase inhibitors in the treatment of gastric cancer.

Drug	Country/Number of Enrollments	NCT number/Phase/Status	Patient population	Treatment arm	Primary efficacy outcome	Treatment-related adverse events	References
Apatinib	China/144	NCT00970138/2,3/Completed	Patients with histologically confirmed GC who were unresponsive or intolerant to at least two prior chemotherapy regimens, including platinum and fluorouracil	Apatinib 850mg vs apatinib 425 mg vs placebo	mOS 2.5 months vs 4.83 months vs 4.27 months, P =0.0017. mPFS 1.4 months vs 3.67 months vs 3.2 months, P < 0.001.	Hypertension, hand-foot syndrome, thrombocytopenia, anemia	(94)
Apatinib, placebo	China/267	NCT01512745/3/Completed	Histologically confirmed GC	Apatinib 850 mg vs placebo	mOS 6.5 months vs 4. 7 months, P=0.0149. mPFS 2.6 months vs 1.8 months, P <0.001.	Leukopenia, neutropenia, hypertension, proteinuria	(95)
Apatinib, paclitaxel, docetaxel	China/321	ChiCTR-OPN-15006601/2/Completed	Patients with cytologically confirmed GC with measurable disease	Apatinib 250mg - 800mg + paclitaxel/docetaxel	mPFS 4.0 months; mOS 8.2 months.	Proteinuria, hypertension, hand-foot syndrome	(96)
Apatinib	China/42	-/2/Completed	Patients with GC who failed second-line chemotherapy or the last chemotherapy failed, no prior molecular targeted therapy	Apatinib 850mg	mFS 4.0 months, mOS 4.5 months.	Secondary hypertension, elevated transaminases	(97)
Apatinib, S-1	China/30	NCT02525237/2/Completed	Histologically confirmed GC	Apatinib + S-1	mPFS 4.21 months, mOS 7.49 months. Patients with lymph node metastasis had prolonged mPFS and mOS when compared with those with liver metastasis (mPFS, 4.21 vs 1.84 months; mOS, 8.21 vs 6.31 months, p = 0.08)	abdominal pain, dizziness, diarrhea	(98)
Apatinib	China/20	NCT02668380/2/Completed	Progressed or recurred GC with prior systemic chemotherapy	Apatinib 850mg	ORR 10%, m OS 4.5 months, mPFS 3.5 months	Hypertension, hand-foot syndrome, anorexia, vomiting, nausea	(99, 100)
Apatinib	China/48	NCT03192735/2/Completed	Endoscopic biopsy-confirmed GC	Apatinib500mg + SOX (S-1: 40-60 mg, oxaliplatin)	R0 resection rate 75.0%.	Neutropenia, leukopenia, elevated transaminases, anemia	(101)
Apatinib	China/48	NCT03104283/2/Completed	Elderly patients with histologically confirmed GC	Apatinib 500mg/250mg	mOS 8.10 months, mFS 3.00 months.	Hypertension, fatigue	(102)
Apatinib	China/337	NCT02668380/2/Completed	Patients with histologically confirmed GC	Apatinib 250mg vs 425-500mg vs 675-850mg	mOS 7.13 months, mPFS 4.20 months.	Hypertension, fatigue, hand-foot syndrome, nausea, proteinuria	(103)
Apatinib Chemotherapy drugs	China/737	NCT03333967/2/Completed	Histologically diagnosed GC	Apatinib monotherapy vs apatinib plus chemotherapy	mOS 8.72 months vs 5.92 months, P < 0.01. mPFS 6.18 months vs 3.52 months, P < 0.01.	Anemia, thrombocytopenia, neutropenia, leukopenia	(104)
Apatinib, Tegafur, Gimeracil, Otrexipotassium	China/126	-/2/Completed	Patients with histologically confirmed GC, inoperable, progressed after systemic chemotherapy	apatinib +s-1 vs s-1	mOS 10.7 months vs 8.1 months, P=0.028. mPFS 5.3 months vs 4.2 months.	Hematological toxicity, vomiting, hypertension, liver and kidney damage	(105)
Apatinib, Tegafur, Gimeracil, Otrexipotassium	China/84	-/2/Completed	Patients with GC who failed second-line and above chemotherapy	Apatinib + s-1	ORR 9.5%, DCR 71.4%	Neutropenia, thrombocytopenia, hypertension, proteinuria	(106)
Apatinib, Tegafur, Gimeracil, Otrexipotassium	China/100	-/2/Completed	Histologically diagnosed GC	Apatinib +s-1 vs s-1	TTP 5.2 ± 0.7 months, OS 9.3 ± 2.5 months, P<0.05	Hypertension	(107)
Apatinib, S -1	China/37	NCT04338438/2/Completed	Patients with GC	Apatinib + S-1	mOS 4.2 months, mPFS 8.2 months.	Hand-foot syndrome, hypertension, diarrhea	(108)
Apatinib, Tegafur, Gimeracil, Otrexipotassium	China/62	-/2/Completed	Patients diagnosed with GC or recurrence after surgery, patients who have received first-line chemotherapy	Apatinib +s-1 vs s-1	mPFS 8.1 months vs 5.0 months, P<0.05.	Hypertension, vomiting, decreased hemoglobin, proteinuria	(109)
Apatinib, Tegafur, Gimeracil, Otrexipotassium, Oxaliplatin	China/39	ChiCTR-ONC-17010430/2/Completed	Patients with untreated unresectable GC	Apatinib +s-1+oxaliplatin	ORR 73.0%, DCR 81.1%	leukopenia, neutropenia	(110)
Apatinib, docetaxel	China/34	-/2/Completed	Histologically diagnosed GC	Apatinib + docetaxel vs apatinib	mOS 6 months vs 3.3 months, P=0.004. mPFS 4 months vs 2.5 months, p=0.002	Leukopenia, neutropenia, anemia, thrombocytopenia	(100)
Apatinib, docetaxel, paclitaxel, tegafur, oxaliplatin, capecitabine	China/32	ChiCTR-OON-1600971/2/Completed	Patients with histologically or cytologically confirmed GC with progression after first-line therapy	Apatinib + chemotherapy	mOS 6.93 months, mPFS 3.06 months.	Hypertension, leukopenia, neutropenia	(111)
Regorafenib, 5- fluorouracil, folinic acid, oxaliplatin	United States/39	NCT01913639/2/Completed	Patients with prior untreated histologically or cytologically confirmed GC	FOLFOX Gary Gorfini vs regorafenib	6 - months PFS rate 53%, ORR 54%	Neutropenia, leukopenia, hypertension	(108)
Regorafenib placebo	Australia/152	ANZCTR 12612000239864/2/Completed	Metastatic or locally recurrent GC	Regorafenib vs placebo	mPFS 2.6 vs 0.9 months	Anorexia, elevated transaminases, abdominal pain, hypertension	(106)
Sorafenib	US/35	NCT00917462/2/Completed	Patients with GC who have progressed on ≤2 prior chemotherapy regimens (or ≤3 prior regimens) in a metastatic setting	Sorafenib	mPFS 3.6 months, mOS 9.7 months	Hand-foot syndrome, vomiting, fatigue, dehydration, hypertension	(112)
Sorafenib, capecitabine, cisplatin	Korea/16	-/1/Completed	GC	Sorafenib, capecitabine, cisplatin	ORR62.5%, m PFS 10 months, m OS 14.7 months.	–	(113)
Oxaliplatin, Sorafenib	Spain/40	-/2/Completed	Patients with GC who have received prior first-line chemotherapy	Oxaliplatin + Sorafenib	mPFS 3 months, mOS 6.5 months.	Neutropenia, thrombocytopenia, neurotoxicity, diarrhea	(114)
Sorafenib, docetaxel, cisplatin	USA/44	NCT00253370/2/Completed	Patients must have measurable, histologically confirmed GC	Sorafenib + docetaxel + cisplatin	ORR 41%, mPFS 5.8 months, mOS 13.6 months.	Neutropenia, hemorrhage at the tumor site	(115)
Sorafenib, 5-fluorouracil	China/46	-/2/Completed	GC	Sorafenib + 5-fluorouracil vs 5-fluorouracil	The 1-year survival rate of the Sorafenib+5-FU group was significantly higher (P<0.05).	–	(116)
Sunitinib	China/78	-/2/Completed	Patients with GC who have received prior chemotherapy	Sunitinib	mOS 6.8 months, mPFS 2.3 months	Neutropenia, thrombocytopenia	(117)
Sunitinib, placebo	Germany/91	NCT01020630/2/Completed	Histologically confirmed GC after the failure of docetaxel and/or platinum-based chemotherapy; FOLFIRI- naïve	Sunitinib + FOLFIRI vs placebo + FOLFIRI	mOS 10.4 vs 8.9 months	Neutropenia, leukopenia	(118)
Sunitinib, irinotecan, fluorouracil, and leucovorin	USA/23	NCT00524186/1/Completed	Histologically confirmed GC or Chemotherapy-naïve patients with GC	Sunitinib + FOLFIRI	mOS 12.4 months, mPFS 6.2months	Anemia, neutropenia, nausea, diarrhea, vomiting, lymphopenia, fatigue	(119)
1Cisplatin, S-1, sunitinib	Japan/27	NCT00553696/1/Completed	Histologically or cytologically confirmed diagnosis of GC	Sunitinib + cisplatin + s-1	ORR 37.5%, m PFS 12.5 months	Neutropenia, leukopenia	(120)
Capecitabine, oxaliplatin, sunitinib, Cisplatin	South Korea/76	NCT00555620 /2/Completed	Patients with GC who have not previously received	Sunitinib + capecitabine/cisplatin or capecitabine/oxaliplatin	mPFS of sunitinib/XP and sunitinib/XELOX was 6.4 months and 5.5-8.0 months; the ORR of sunitinib/XP and sunitinib/XELOX was 46.7% and 43.5-45.5%.	Nausea, stomatitis, hypophosphatemia	(121)
Pazopanib + capecitabine + oxaliplatin	South Korea/66	NCT01130805/2/Completed	Patients with histologically confirmed unresectable metastatic or recurrent GC	Pazopanib + capecitabine + oxaliplatin	ORR 62.4%, mPFS 6.5 months, mOS 10.5 months.	Neutropenia, anemia, thrombocytopenia, anorexia, nausea, vomiting	(122)
Pazopanib, 5-fluorouracil, folinic acid, and oxaliplatin	Germany/75	NCT01503372/2/Completed	Patients with histologically confirmed GC, surgically incurable and chemotherapy-naïve.	Pazopanib + FLO vs FLO	6-month PFS rate 34% vs 30%, mPFS 4.66 vs 4.47 months, mOS 10.19 vs 7.33 months, ORR 72% vs 59%	Loss of appetite, nausea, fatigue, diarrhea, neutropenia, thrombocytopenia	(123)

R0, complete tumor resection with negative margins under the microscope, good prognosis; s-1, Tegafur Gimeracil Oteracil Potassium; SOX, oxaliplatin + S-1; FOLFIRI, irinotecan + 5-fluorouracil + calcium folinate; FOLFOX, 5-fluorouracil; calcium folinate, oxaliplatin; FLO, 5-fluorouracil + oxaliplatin.

Apatinib is mainly used for advanced GC patients who have failed chemotherapy (124). The earliest study of apatinib was a phase II trial for patients with metastatic GC. A total of 144 patients with GC failed in second-line or more chemotherapy was enrolled, of which the apatinib components were 850mg/qd and 425mg/bid. The results showed that both mPFS and mOS were improved, and there were significant statistical differences between apatinib and placebo (94). A randomized, double-blind phase III clinical study of apatinib (95)has shown that for patients with GC for whom two or more prior chemotherapy regimens failed, mOS and mPFS were significantly improved in apatinib group compared with placebo. These suggested that apatinib treatment significantly improved the OS and PFS of patients with GC resistant to two or more prior chemotherapy regimens and increased the survival time of patients. These two trials had consistent conclusions regarding adverse reactions. The most common grade 3 to 4 non-hematological adverse events were hand-foot syndrome, proteinuria, and hypertension. Most patients could tolerate and safety was acceptable. Studies by Shen (96)and Ruan (97) also showed that apatinib had good efficacy and safety in patients with GC irresponsive to two or more prior chemotherapy regimens.

Subsequent studies compared the efficacy of different doses of apatinib on the survival of patients with advanced GC, and the results showed that compared with higher daily doses (675-850mg) of apatinib, lower daily doses (250-500 mg) of apatinib can achieve comparable outcomes in mOS and mPFS while maintaining more benign safety profile (99, 103, 104) in patients with GC. However, the efficacy and safety of apatinib in elderly patients with GC remain unclear, so an open-label, single-arm, phase II study was conducted involving GC patients aged ≥60 years (48 patients). Results showed that apatinib was effective and relatively well-tolerated in elderly patients with unresectable GC who had received at least one line of chemotherapy (102), and a lower initial daily dose (250mg–500mg) may be an appropriate choice for elderly patients in clinical practice (127).

Chemotherapy alone has limited benefit in patients with GC who have failed first-line therapy. Therefore, exploring which chemotherapy regimens can effectively prolong their survival and improve the quality of life by combining apatinib has become a current research focus for advanced GC patients who have failed chemotherapy. In the clinical trials of treatment of GC with apatinib combined with docetaxel, the mPFS and mOS of the apatinib group and the combination group were 2.5 and 4 months, 3.3 and 6 months, respectively, and grade 3/4 adverse reactions such as neutrophils, cytopenia, anemia, thrombocytopenia, and hypertension were milder in the combination group than in the apatinib group (128). Patients with advanced GC benefited more from apatinib plus docetaxel compared with apatinib monotherapy (129). Apatinib plus docetaxel was proved clinically beneficial in previous studies, but the feasibility of combining apatinib with other chemotherapeutic agents was unclear.

Subsequent studies have found that apatinib combined with chemotherapy has also achieved a good curative effect in the second-line treatment of AGC. Apatinib combined with chemotherapy as the second-line treatment of advanced GC has good clinical efficacy and acceptable side effects, and may provide a new second-line treatment option for patients with advanced GC (130). Apatinib and s-1 (tegafur + gimeracil + oteracil potassium) have been approved by the National Medical Products Administration (NMPA) of China for the treatment of GC, and patients can afford these treatments (131). Meanwhile, manageable adverse events reduced the side-effect costs of symptomatic and supportive care (129). Apatinib combined with s-1 therapy was superior to s-1 alone in the second-line treatment of GC. The combination can significantly improve the quality of life of patients, reduce the level of serum tumor markers, prolong the patient’s mOS (105), and mPFS, and improve ORR and disease control rate (DCR) (106). In another study, apatinib also improved the levels of T helper 1 (Th1) and T helper 2 (Th2)-like cytokines (107). Apatinib in combination with S-1 has shown promising efficacy and manageable toxicity as a second-line treatment for patients with GC, especially for elderly patients with poor performance status (108). Combination therapy with apatinib, especially with paclitaxel, may confer a better survival benefit in the first-line treatment (132). However, some studies have also suggested that while increasing the curative effect, combined drug use reduced the quality of life of patients and increased the risk of adverse reactions (109).

Apatinib has also demonstrated certain therapeutic effects targeting metastatic gastric cancer in clinical trials. Apatinib combined with S-1 as a first-line treatment for GC was not superior to other chemotherapy regimens. Toxicities were consistent with known profiles when given as monotherapy (131). Notably, this study compared metastatic sites in GC. Compared with patients with liver metastases, patients with lymph node metastases gained better curative effects as they tended to have prolonged mPFS and mOS. This may support the design of future clinical trials to better define patient populations (98). The study also reported that the most common grade 3 to 4 AEs for apatinib monotherapy were hypertension, hand-foot syndrome, anorexia, vomiting, and nausea. Apatinib combined with SOX (S-1+oxaliplatin) as a neoadjuvant therapy for advanced or metastatic GC also has demonstrated significant efficacy and safety and the common adverse reactions include leukopenia, neutropenia and hypertension. Further randomized clinical trials at a larger scale are needed to confirm these findings (101, 110). Apatinib showed promising efficacy and acceptable safety in GC patients with advanced liver metastases. Anti-angiogenic therapy may be a good strategy for the treatment of GC with liver metastases, a rare subtype of GC (100).

### 4.2 Fruquintinib

Fruquintinib is an orally available, highly selective small-molecule antagonist of VEGFR1, VEGFR2, and VEGFR3 (111). In September 2018, fruquintinib received its first global approval in China for the treatment of metastatic colorectal cancer in patients who have failed at least two prior systemic antineoplastic treatments (133). Currently, there are ongoing phase 2 and phase 3 studies (NCT02415023, NCT03223376) of fruquintinib combined with paclitaxel in the treatment of GC, and phase 2 trials of fruquintinib combined with SOX (NCT05122091) as neoadjuvant therapy for GC are also underway (111). The phase III clinical development of fruquintinib monotherapy is mainly for patients with advanced non-small cell lung cancer (NSCL) and GC (134)(**Table 2** for more details).

### 4.3 Sorafenib

Sorafenib has been shown to have inhibitory effects against platelet-derived growth factor receptor (PDGFR), VEGFR2, VEGFR-3, PDGFR-β and other receptors (135). It has dual anti-tumor effects (136). On the one hand, it can block the formation of tumor angiogenesis by inhibiting VEGFR and PDGFR to indirectly inhibit the growth of tumor cells (137). On the other hand, it can directly inhibit tumor growth by blocking the RAF/MEK/ERK signaling pathway (138).

The results of the phase II clinical study (NCT00917462) showed (112) that single-agent sorafenib can improve OS and PFS in patients with advanced gastroesophageal junction adenocarcinoma. Treatment-related adverse reactions include hand-foot syndrome, rash, dehydration and fatigue, and mutations of P53 and other related gene identified by tumor exome sequencing. This may bring new opportunities for sorafenib in the treatment of gastroesophageal junction adenocarcinoma (139). Sorafenib can also be used in combination with various chemotherapy drugs, including paclitaxel, cisplatin, and 5- fluorouracil. Subsequent studies have showed that the triple combination of sorafenib, docetaxel, and cisplatin had clinical activity. There are few works of literature on the maximum tolerated dose of sorafenib combined with chemotherapy drugs. A phase I trial once mentioned sorafenib (400mg/bid), capecitabine (800mg/m2/bid) and cisplatin (60mg/m2) were recommended as a first-line treatment in GC (113). In addition, a phase II trial demonstrated the efficacy of sorafenib in combination with docetaxel and cisplatin for the treatment of advanced GC, with an mOS of 13.6 months (114) and the most common grade 3/4 adverse reactions being neutropenia. In a phase I study of sorafenib in combination with S-1 and cisplatin for the treatment of advanced GC, pharmacokinetic analysis showed no significant difference in the sorafenib exposure between the sorafenib group and combination group, with adverse reactions including anorexia, rash, neutropenia, thrombocytopenia, and nausea (115). Sorafenib in combination with 5-FU can effectively decrease serum VEGF and HIF-1α levels and improve 1-year survival rate (116). In a trial of sorafenib in combination with oxaliplatin as a second-line treatment for advanced GC, the mPFS was 3 months and the mOS was 6.5 months. However, subgroup analyses of this trial showed that the progression-free time of first-line treatment determined the different prognosis of patients, and the grade 3/4 adverse reactions were neutropenia and thrombocytopenia (140).

From the above, it can be seen that sorafenib alone or in combination with different chemotherapy drugs can be used for the treatment of advanced GC, but results of high-quality trials are needed to support the viewpoint. Therefore, more in-depth research on the use of sorafenib in the treatment of advanced gastric cancer should be carried out. Meanwhile, dose change of sorafenib was in correlation with the occurrence of adverse events (141), so the combination of sorafenib with chemotherapy drugs should be further explored in large-scale cohort studies. (**Table 2** for more details).

### 4.4 Sunitinib

Sunitinib is also a multi-target tyrosine kinase inhibitor against VEGF and PDGFR-β (142). Sunitinib monotherapy (117)was tolerated in GC, but tumor responses were limited. Although sunitinib monotherapy only has insufficient clinical value as a second-line treatment for GC, its role in combination with chemotherapy deserves further study (143). Later studies found that sunitinib combined with FOLFIRI tended to improve the overall survival of GC (118, 119), but the primary endpoint was not reached. Therefore, the clinical efficacy of sunitinib in patients with GC who failed first-line treatment is not satisfactory. Phase I dose trial suggested that sunitinib plus cisplatin 80 mg/m2 and 5-FU 4,000 mg/m2 were combinable with controllable adverse events (144), and the maximum tolerated dose of sunitinib (MTD) was determined to be 25 mg/day. A Japanese clinical study (120) showed that in a phase I trial of sunitinib combined with s-1 and cisplatin in patients with GC, the MTD of sunitinib combined with cisplatin/S-1 was 25 mg/day. The regimen showed a manageable safety profile and preliminary antitumor activity. Among Korean patients, sunitinib combined with XELOX (oxaliplatin + capecitabine) in patients with advanced GC (121) had an mPFS of 5.5-8.0 months and an ORR of 43.5-45.5%. This suggests that sunitinib has shown good safety in Asian countries such as Japan and South Korea, with relatively consistent tolerated doses (**Table 2** for more details).

### 4.5 Pazopanib

Pazopanib is an orally available and selective tyrosine kinase inhibitor against targets such as VEGFR-1/-2/-3 and PDGFR to inhibit angiogenesis, which has been approved for advanced kidney cancer and soft tissue sarcoma treatment (145, 146). Kim et al. designed a single-arm, open-label phase II study (122) to determine the efficacy and toxicity of pazopanib plus XELOX in GC treatment. The published results of the study indicated that the combination showed moderate activity and an acceptable toxicity profile in patients with GC. The main adverse reactions of grade 3 or above were neutropenia, anemia, thrombocytopenia, and loss of appetite. Subsequent case reports suggested that pazopanib alone can produce sustained efficacy in recurrent and metastatic gastroesophageal adenocarcinoma (147). An open-label randomized phase II trial (123) (2:1) investigated the efficacy of pazopanib plus FLO (5-fluorouracil + oxaliplatin) versus FLO monotherapy as first-line therapy in patients with GC. The results indicated that adding pazopanib to chemotherapy showed signs of efficacy, but no significant improvement. The combination was well tolerated but had high toxicity, and the main adverse events included loss of appetite, nausea, and fatigue (**Table 2** for more details).

## 5 Response biomarkers of anti-angiogenic drug therapy

Anti-angiogenic drugs mainly act on vascular epidermal growth factor, and have shown curative effects in most clinical trials to prolong the survival time of some patients with GC (148). Many clinical studies showed the potential efficacy benefits of anti-angiogenic drugs and their combination therapy, but there are still challenges (149). Determining which patients can get the most benefit from this treatment is the top challenge and it requires specific biomarkers for screening. The following summary expands on response biomarkers (Details in **Table 3**).

**Table 3:** Predictive response biomarkers for anti-angiogenic targeted therapy in gastric cancer.

Intervention	Name and Conclusion of Predictive Biomarkers	References
Ramucirumab	VEGFR-2	(35)
FOLFIRI plus ramucirumab	28 of 29 patients (96.6%) in the FOLFIRI plus ramucirumab group underwent genomic analysis. All patients with available results (next-generation sequencing and/or IHC) were microsatellite stable and 20% (4/20 tests) were PD - L1 positive	(69)
Ramucirumab + Paclitaxel	The function of 3 angiogenesis-related mediators, such as VEGF-A, VEGF-D, and sVEGFR-2, as potential prognostic and predictive biomarkers in metastatic GC treated with second-line paclitaxel plus ramucirumab. We reported an association between higher baseline levels of VEGF-A and shorter OS. We also found an association between elevated sVEGFR-2 levels after 1 cycle and prolonged PFS and OS.	(150)
Bevacizumab + chemotherapy	Plasma VEGF-A and tumor neuropilin-1 were strong candidate biomarkers for predicting clinical outcome in patients with GC treated with bevacizumab	(151)
Apatinib	VEGFR2	(95)
Apatinib+s-1	Serum carbohydrate antigen 19-9 (CA19-9), CEA, and tumor supply group factor (TSGF) levels were significantly reduced	(105)
Apatinib+s-1	CEA, CA199 and carbohydrate antigen 125 (CA125) were significantly reduced, reducing interferon- Gamma (IFN-γ), TNF -α, Interleukin-4 (IL-4) and Interleukin - 10 (IL-10) (P<0.05)	(107)
Apatinib+s-1	TP53 was the most common mutation (18/25), CDH1 and APC were the second most common (5/25).	(98)
Apatinib + Chemotherapy	Early-onset anti-angiogenic-related AEs, including hypertension, proteinuria, or hand-foot syndrome, were viable biomarkers of antitumor efficacy in patients with metastatic GC	(132)
Apatinib	CEA was considered a potential independent predictor associated with shorter PFS and OS.	(100)
Regorafenib + FOLFOX	Six patients with ERBB2 amplification benefited from regorafenib plus FOLFOX. By targeting multiple tyrosine kinases, regorafenib blocked RTK-RAS-PI3K signaling, which was overactivated in HER2-positive tumors.	(108)
Regorafenib	The benefit of regorafenib was comparable in patients with VEGF-A levels above and below the median.	(106)
Sorafenib	Whole-exome sequencing of this tumor revealed mutations in many cancer-associated genes, including ARID1A, PIK3CA, and TP53, as well as local amplifications of HMGA2 and MET.	(112)
Sorafenib	Tumor cells can proliferate under hypoxic conditions, which is closely related to the activation of hypoxia-inducible factor-1α (HIF-1α) and vascular endothelial growth factor (VEGF). HIF-1α can enhance cell metabolism under hypoxic conditions and contribute to the activation of VEGF to induce tumor angiogenesis. HIF-1α expression may be a predictor of poor prognosis in GC, especially in Asia.	(152)
Sorafenib +5-FU	Chemotherapy combined with sorafenib can effectively reduce serum HIF-1α and VEGF levels in patients with GC and improve their 1-year survival rate and prognosis.	(116)
Sunitinib	There was a modest association between elevated baseline plasma VEGF-C levels and above-median OS (P = 0. 0241).	(117)
Sunitinib + FOLFIRI	In the subgroup serum analysis, significant changes in serum levels of VEGF-A (P = 0.017), VEGFR2 (P = 0.012) and VEGF-D (P < 0.001) were observed.	(118)
Sunitinib	Tumor VEGF-C expression (vs non-expression) was associated with significantly shorter median PFS in a subgroup of sunitinib monotherapy trials of patients with GC; no difference in tumor control rate	(153)
Pazopanib + capecitabine + oxaliplatin	FGFR2 expression checked by immunohistochemistry may be a useful biomarker for predicting metastatic or recurrent GC patients receiving pazopanib combined with CapeOx	(154)

At present, anti-angiogenic drugs have obtained positive results in the treatment of GC, but the discovered response biomarkers have not been verified as predictive or prognostic. Biomarkers in tumor tissue or the circulation of cancer patients may serve as response biomarkers (155). As mentioned earlier, in GC, ramucirumab alone or its combination with paclitaxel as second-line therapy has survival benefits. Although VEGF-D is a potential biomarker for ramucirumab in colorectal and hepatocellular carcinoma, earlier studies did not identify it as a useful biomarker for patients with GC (156). Later studies (150) evaluated the possibility of VEGF-A, VEGF-D and soluble vascular endothelial cell growth factor receptor-2 (sVEGFR-2) serving as the response biomarkers of resistance or efficacy in ramucirumab and paclitaxel combination. The results showed an association between higher baseline levels of VEGF-A and shorter OS, and there was an association between elevated sVEGFR-2 after one week and prolonged PFS and OS. This was also the first report supporting sVEGFR-2 as a positive marker after treating metastatic GC with the combination of paclitaxel and ramucirumab. Ramucirumab binds to VEGFR2 on vascular endothelial cells to inhibit VEGF ligand binding and receptor signaling and limit VEGF-induced angiogenesis and endothelial cell migration, thus slowing tumor growth (157). VEGFR-2 signaling was an important therapeutic target in GC (35). GC with VEGFR-2 overexpression have a poor prognosis, indicating VEGFR-2 may be a negative prognostic marker (158). However, the REGARD trial analysis found that the prognostic trend between high VEGFR-2 endothelial expression and shortened progression-free survival was not significant. Further studies are needed to investigate the predictive potential of high VEGFR2 expression in patients with GC treated with ramucirumab (159). In addition to predictable biomarkers in the VEGF family, patients in a ramucirumab plus FOLFIRI arm who underwent genomic analysis were all microsatellite stable and programmed death ligand 1 (PD -L1) may be a potential positive prognostic marker (69).

The addition of bevacizumab to chemotherapy improved progression-free survival and tumor response rates of patients with GC, but overall survival was not affected. To test the hypothesis that angiogenic markers might have predictive value for the efficacy of bevacizumab in GC, AVAGAST included a prospective, mandatory biomarker program (151). Plasma was available from 712 patients (92%) and tumor samples were available from 727 patients (94%). Baseline plasma VEGF-A levels and neuropilin-1 expression were identified as potential predictors of bevacizumab efficacy (160).

Currently, the analysis of biomarkers of apatinib in the treatment of GC after chemotherapy is mainly based on serum VEGFR-2 (95), serum carbohydrate antigen 19-9, carcinoembryonic antigen (CEA), tumor supplied group factor (TSGF), tumor necrosis factor- α (TNF-α) and inflammatory factors (105, 107). It has also been suggested (98) that TP53 was the most commonly mutated gene, with CDH1 and APC genes being the second most common. Early anti-angiogenesis-related adverse events, such as hypertension, proteinuria, and hand-foot syndrome (120.126), were listed as feasible biomarkers of efficacy.

Preliminary biomarker analysis of the INTEGRATE trial showed a similar benefit of regorafenib in patients with VEGF-A levels above and below the median (106). The ERBB2 gene was also predicted to be a viable efficacy biomarker, as this gene amplification benefited from regorafenib plus FOLFOX treatment.

Sorafenib is used for the treatment of gastrointestinal stromal tumors and metastatic renal cell carcinoma in patients who do not respond to or cannot tolerate standard therapies (161). Sorafenib can selectively target certain proteins to regulate tumor cell growth and metabolism (162). Tumor cells can proliferate under hypoxic conditions and this is closely related to the activation of hypoxia-inducible factor-1α (HIF-1α) and VEGF. HIF-1α can enhance cell metabolism under hypoxic conditions and contribute to the activation of VEGF to induce tumor angiogenesis. HIF-1α expression may be a predictor of poor prognosis in GC, especially in Asia (152). Chemotherapy combined with sorafenib can effectively reduce serum HIF-1α and VEGF levels in patients with GC to improve their 1-year survival rate and prognosis (116). Diffuse expression of HIF-1α in gastric tumors may lead to resistance to adjuvant chemotherapy with 5-FU (163). Local amplification of associated genes ARID1A, PIK3CA, and P53, as well as HMGA2 and MET, also benefited from sorafenib treatment (112).

Tumor VEGF-C expression (compared with no expression) was associated with significantly shorter median PFS and above-median OS in a subgroup of sunitinib monotherapy trials of patients with GC, but tumor control rates did not differ (153). Serum VEGF-A, VEGFR2 and VEGF-D have also been shown to be sensitive to this therapy (118).

FGFR2 gene expression by immunohistochemistry may be a useful biomarker for predicting patients with metastatic or recurrent advanced GC to receive pazopanib combined with CapeOx (154).

## 6 Challenges and solutions of anti-angiogenic targeted therapy for GC

Anti-angiogenic drugs target various aspects of tumor angiogenesis to block the formation of blood vessels to cut off the nutrient and supply to tumor cells, resulting in a hypoxic microenvironment (164). This therapy has shown limited efficacy, with survival benefits ranging from weeks to several months (165), which may be related to the fact that tumors can activate alternative pathways of angiogenesis, increase invasiveness and metastasis, or develop resistance to anti-angiogenic therapy by immune system inhibition (166). Tumor vessels display tortuosity, disorganization, leakiness, slow blood flow, and hypoxia (167), thus early use of anti-angiogenic drugs can improve the “chaotic” state and normalize gastric tumor vessels (168). However, the early balance of pro-angiogenic and anti-angiogenic factors is temporary (169). Although there has been some progress in anti-angiogenic therapy for GC, the survival benefits of this treatment still face many challenges.

(1) Anti-angiogenic therapy can lead to hypoxia-induced apoptosis of tumor cells while cloning hypoxia-resistant tumor cells, which can lead to drug resistance (170). HIF-1α induces epithelial-mesenchymal transition (EMT), which further enhances the ability of tumor cells to tolerate hypoxia, locally invade, infiltrate blood vessels, and survive in peripheral blood vessels (171). Experimental studies on gastric cancer cell lines CUM-2MD3 and OCUM-12 have found that under hypoxic conditions, GC cells form EMT through autocrine stimulation of TGFβ factors (172). The EMT cell transcriptome is characterized by the expression of proteins with multiple functions, such as growth factors and corresponding protein factor receptors (TGFβ, HGF, HGFR), accessory transcription factors (Wnt, Notch, NFkB), integrin receptors, proteoglycan joint receptors CD44, and glucose-6-phosphate isomerase (GPI) (173). Among these factors, Notch and Wnt are closely related to the HIF-1α signaling pathway (174).
(2) The hypoxic state induces and recruits bone marrow cells to assist in tumor-induced neovascularization (175). Under hypoxic conditions, HIF-1α and its targets, Stromal cell-derived factor-1 (SDF-1) and VEGF will increase to attract a heterogeneous group of bone marrow-derived cells composed of vascular progenitor cells and pro-angiogenic monocytes (176). Endothelial and pericyte progenitor cells are bound as part of the new blood vessels to directly build new blood vessels (177). Pro-angiogenic monocytes provide energy to the tumor by producing pro-angiogenic cytokines, growth factors, and proteases (178). All of these contribute to the formation of new blood vessels.
(3) When tumors become hypoxic, compensatory pathways can be activated to circumvent anti-angiogenic therapy by switching to different pro-angiogenic factors that lead to neovascularization and upregulation of tumor invasiveness, ultimately resulting in tumor recurrence (179). Studies have shown (180) that continuous use of anti-angiogenic drugs can enhance hypoxia and induce upregulation of other factors associated with angiogenesis, such as PIGF, fibroblast growth factors (FGFs), and inflammatory chemokines. This can also recruit Tie2-expressing monocytes (TEMs) and tumor-associated macrophages (TAMs) to promote angiogenesis and disrupt the temporary balance achieved by anti-angiogenic therapy, leading to chaotic and disordered growth of tumor vasculature (181).
(4) TKI can increase vascular permeability and lead to the hematogenous metastasis of cancer cells when it disrupts tumor vascular stability (182). The migration of tumor cells towards blood vessels is influenced by tumor-associated macrophages (TAMs), which can stimulate tumor angiogenesis, making tumor vessels more chaotic. Meanwhile, TAMs secrete EGF and stimulate the EGFR to strengthen the invasive ability of tumors (183). The increase of the expression of matrix metalloproteinases (MMPs), urokinase-type plasminogen activator receptor (uPAR), and tissue proteases will improve the vascular permeability, making tumor cells more prone to intravasation (184).
(5) Vasculogenic mimicry (VM) is the formation of vessel-like channels supplying blood to tumor tissue, which is achieved through the interaction between tumor cells and the extracellular matrix (185). It is also the result of high expression of HIF-1α in tumor cells.
(6) VEGF and its signaling pathway inhibit the maturation of dendritic cells and induce the development of regulatory T cells in the tumor microenvironment. VEGF also promotes the expression of PD-1 in tumor cells, which leads to T cell exhaustion and ultimately destroys the anti-tumor immune response, resulting in immune cell inhibition (186).

There are available solutions to the above issues. Firstly, we have to find the treatment regimens of combination therapy with anti-angiogenic drugs. The combination of anti-angiogenic drugs and chemotherapy drug docetaxel can effectively inhibit the synthesis of mitochondrial DNA in hypoxic tumor stem cells, thereby improving the efficacy of anti-angiogenic drugs (187). There are also studies on the combination of anti-angiogenic drugs with other targeted drugs, such as the combination of bevacizumab and trastuzumab to improve mPFS and mOS in advanced GC patients (188). The combination of anti-angiogenic therapy and immunotherapy has also achieved certain effectiveness, such as the combination of bevacizumab and PD-1 inhibitor (189). Nanoparticles have also shown the ability to target endothelial cells, effectively delivering anti-angiogenic drugs to the tumor site and enhancing the therapeutic effect by reducing systemic toxicity (190).This therapy is still at an early stage of development and shows great potential in inhibiting tumor angiogenesis (191).

## 7 Discussion

Anti-angiogenic targeted therapy aims to block the formation of new blood vessels that nourish tumors. Anti-angiogenic therapy may have some advantages in GC treatment because patients with this type of cancer have high levels of VEGF, a key factor that stimulates angiogenesis (192).

This review summarizes current clinical trials and response biomarkers of anti-angiogenic targeted therapy for GC with complete data published in open-access journals. We first summarize the efficacy of the reported clinical trials of anti-angiogenic targeted therapy drugs in patients with GC. The clinical trials of ramucirumab were large-scale, involving multiple continents and countries, and it has been proved that single drug or ramucirumab paclitaxel combination in the treatment of GC can prolong the PFS and OS of patients, thus becoming the standard second-line therapy for GC. Bevacizumab was first approved for the treatment of metastatic colorectal cancer. Compared with ramucirumab (158), its single-drug effect was not obvious and clinical trials were not sufficient. Later studies found that bevacizumab combined with conventional chemotherapy can improve the curative effect and prolong OS in patients with GC. Apatinib tyrosine kinase inhibitors entered clinical trials to test GC at the beginning, and it showed good efficacy in patients with GC after they failed chemotherapy. Subsequent studies confirmed that low-dose apatinib was more effective and safer for patients with GC who have received prior extensive treatment. Apatinib is orally available and that is conducive to clinical promotion (159). Sorafenib has proven clinical activity in clinical trials, but sunitinib, pazopanib and fruquintinib have been less consistent. Most of the response biomarkers of anti-angiogenic targeted therapy have not been verified.

We also found the characteristics of clinical trials of anti-angiogenic targeted therapy. There were only a few single drug interventions in the clinical trials summarized in this article as most of the trials were drugs combined with chemotherapy. Besides, most studies were single-arm trials, and the research endpoints were mainly ORR, mPFS, mOS, and side effects. However, there were also problems. First, there were many types of anti-angiogenic targeted drugs, while clinical trials of single drugs were limited, and trials with positive results were even fewer. Secondly, there were many combined treatment options for anti-angiogenic targeted drugs and some were still in the initial stage. Third, the efficacy of these drugs was different in different patient groups, but the subgroup analysis for this issue was not comprehensive. Fourth, only a few drugs in the review have undergone clinical trials of the optimal dose selection. Fifth, the discovery of response biomarkers in this paper was not deep enough, and the main prediction was limited to response markers. Many biomarkers have been reported but they have not been widely used in clinical practice, thus the overall predictive efficiency and level of evidence were low. It can be seen that anti-angiogenic therapy cannot cure gastric cancer nor is it effective for all patients. It may only benefit a subset of patients with certain molecular features or biomarkers that predict response to the therapy (193). Over time, however, anti-angiogenic therapy may lead to resistance or relapse as tumors adapt to the lack of blood supply (194).

Future clinical research on anti-angiogenic targeted drugs can focus on the following directions: First, when selecting anti-angiogenic targeted drugs, researchers should pay attention to the stage and type of GC of the patient, and continue to explore the best time for applying the drugs for specific GC. Secondly, pay attention to the design of the intervention arm and the establishment of research endpoints in designing clinical trials. Thirdly, pay attention to the detailed subgroup analysis (race, age, different centers, etc.). Fourthly, pay attention to the selection of the optimal dose, index setting and dose tolerance for curative effect evaluation. Fifth, explore the efficacy of anti-angiogenic targeted drugs combined with different chemotherapeutic drugs or different types of targeted drugs for better curative effects and less toxic and side effects. Sixth, the verification of biomarkers still needs a large number of prospective studies (efficacy, toxicity, and drug resistance).

## Author contributions

DX, YL designed the study together, equal contribution, Listed as co-first author. GF, PY as co-corresponding author, PW, JL, LM, JH, HZ, XY, LL and YZ, were all involved in the revision of the manuscript. GF and PY made final critical revisions. All authors contributed to the article and approved the submitted version.
